# Supplementary material for: Transfer learning with convolutional neural networks for cancer survival prediction using gene-expression data
Source: PLoS One. 2020 Mar 26;15(3):e0230536. doi: 10.1371/journal.pone.0230536 (PMC7098575; doi:10.1371/journal.pone.0230536)
Supplement: S1 Table — (PDF) [file pone.0230536.s001.pdf]

Table 1. CNN hyper-parameters optimization.

**Category**	**Hyper-parameter**	**Search space**
Convolutional Layer 1	Number of filters	$\{2, 4, 8, 12, 16\}$
	Kernel size	$\{4 \times 4, 8 \times 8, 12 \times 12, 16 \times 16, 20 \times 20\}$
	Dropout	$\{0.2, 0.4, 0.6, 0.8\}$
Convolutional Layer 2	Number of filters	$\{8, 12, 16, 32, 40\}$
	Kernel size	$\{2 \times 2, 4 \times 4, 8 \times 8, 12 \times 12, 16 \times 16\}$
	Dropout	$\{0.2, 0.4, 0.6, 0.8\}$
Fully-connected Layer 1	Number of units	$\{120, 160, 200, 240\}$
	Dropout	$\{0.2, 0.4, 0.6, 0.8\}$
Fully-connected Layer 2	Number of units	$\{25, 50, 75, 100\}$
	Dropout	$\{0.2, 0.4, 0.6, 0.8\}$
Architecture	Number of FC layers	$\{1, 2\}$
Pre-training	Resampling ratio	{1:1, 2:1, 3:1, 4:1}
	Learning rate	$\log U(1 \times 10^{-3}, 1 \times 10^{-1})$
	Batch size	$\{64, 128, 256, 384, 512\}$
Fine-tuning	Resampling ratio	{1:1, 2:1, 3:1, 4:1}
	Learning rate	$\log U(5 \times 10^{-4}, 1 \times 10^{-1})$
	Batch size	$\{32, 80, 128, 192, 256\}$
