# Supplementary material for: Transfer learning with convolutional neural networks for cancer survival prediction using gene-expression data
Source: PLoS One. 2020 Mar 26;15(3):e0230536. doi: 10.1371/journal.pone.0230536 (PMC7098575; doi:10.1371/journal.pone.0230536)
Supplement: S2 Table — (PDF) [file pone.0230536.s002.pdf]

Table 2. MLNN hyper-parameters optimization.

Category	Hyper-parameter	Search space
Hidden Layer 1	Number of units	$\{1500, 1750, 2000, 2250, 2500, 2700\}$
	Dropout	$\{0.2, 0.4, 0.6, 0.8\}$
Hidden Layer 2	Number of units	$\{200, 400, 700, 1000\}$
	Dropout	$\{0.2, 0.4, 0.6, 0.8\}$
Hidden Layer 3	Number of units	$\{30, 80, 120, 160\}$
	Dropout	$\{0.2, 0.4, 0.6, 0.8\}$
Architecture	Number of hidden layers	$\{2, 3\}$
Pre-training	Learning rate	$\log U(1 \times 10^{-3}, 1 \times 10^{-1})$
	Batch size	$\{64, 128, 256, 384, 512\}$
	Resampling ratio	{1:1, 2:1, 3:1, 4:1}
Fine-tuning	Learning rate	$\log U(5 \times 10^{-4}, 1 \times 10^{-1})$
	Batch size	$\{32, 80, 128, 192, 256\}$
	Resampling ratio	{1:1, 2:1, 3:1, 4:1}
