# Supplementary material for: Transfer learning with convolutional neural networks for cancer survival prediction using gene-expression data
Source: PLoS One. 2020 Mar 26;15(3):e0230536. doi: 10.1371/journal.pone.0230536 (PMC7098575; doi:10.1371/journal.pone.0230536)
Supplement: S3 Table — (PDF) [file pone.0230536.s003.pdf]

Table 3. ML models hyper-parameters optimization.

Category	Hyper-parameter	Search space
Feature selection	Number of variables	$\{150, 200, 250, 300, 350\}$
Feature extraction	Number of components	$\{150, 200, 250, 300, 350\}$
SMOTE	Resampling ratio	{1:1, 2:1, 3:1, 4:1}
	K-neighbours	$\{3, 5, 7, 9\}$
LR	Regularization	$\{L1, L2\}$
	C	$\log U(1 \times 10^{-4}, 1 \times 10^{3})$
	Maximum iterations	$\{1 \times 10^{4}, 1 \times 10^{5}, 1 \times 10^{6}, 1 \times 10^{7}\}$
SVM	Gamma	$\log U(1 \times 10^{-4}, 1 \times 10^{3})$
	C	$\log U(1 \times 10^{-4}, 1 \times 10^{3})$
	Maximum iterations	$\{1 \times 10^{4}, 1 \times 10^{5}, 1 \times 10^{6}, 1 \times 10^{7}\}$
NN	Number of hidden units	$\{25, 50, 75, 100\}$
	L2 regularization	$\log U(1 \times 10^{-6}, 1 \times 10^{-1})$
	Learning rate	$\log U(5 \times 10^{-5}, 1 \times 10^{-1})$
	Batch size	$\{20, 50, 80, 110, 140, 170\}$
RF	Number of trees	$\{50, 100, 300, 500, 700\}$
	Maximum depth	$\{10, 30, 50, 70, 90\}$
	Minimum samples split	$\{0.05, 0.1, 0.15, 0.2, 0.3\}$
	Minimum samples leaf	$\{0.03, 0.06, 0.1, 0.2\}$
