# Supplementary material for: Transfer learning with convolutional neural networks for cancer survival prediction using gene-expression data
Source: PLoS One. 2020 Mar 26;15(3):e0230536. doi: 10.1371/journal.pone.0230536 (PMC7098575; doi:10.1371/journal.pone.0230536)
Supplement: S1 File — (PDF) [file pone.0230536.s004.pdf]

Supporting information - Transfer learning with convolutional neural networks for cancer survival prediction using gene-expression data

Guillermo López-García, José M. Jerez, Leonardo Franco, and Francisco J. Veredas

Departamento de Lenguajes y Ciencias de la Computación, Universidad de Málaga, ETSI Informática, Málaga, Spain

1 Code and reproducibility

For reproducibility of the results obtained in this paper, all the code and data used in this work is publicly available at `https://github.com/guilopgar/GeneExpImgTL`, including the Jupyter notebooks implemented to generate the KEGG BRITE functional hierarchies structure, obtain the gene-expression images, train and evaluate the models, etc. We hope that all the effort made to make these resources public will promote further research on using advanced computational methods to contribute to the progress of precision medicine based on the analysis of gene-expression data.

2 Hyper-parameter optimization

Here, we describe the hyper-parameter space used by the Bayesian procedure when optimizing the hyper-parameters of each model. In this way, Table 1 describes the hyper-parameters optimization of the convolutional neural network (CNN) model, which is trained following a transfer learning (TL) approach. In Table 2, the hyper-parameters optimization procedure of the multi-layer neural network (MLNN)—which is also trained following a TL approach—is depicted. Finally, in Table 3, the tuning of the whole set of hyper-parameters included in the traditional machine learning (ML) approaches is described. This set contains the configuration parameters of the feature selection/extraction methods, synthetic minority over-sampling technique (SMOTE), logistic regression (LR), support vector machines (SVM), neural networks (NN) and random forest (RF) classifiers.
