# Supplementary material for: Transfer learning with convolutional neural networks for cancer survival prediction using gene-expression data
Source: PLoS One. 2020 Mar 26;15(3):e0230536. doi: 10.1371/journal.pone.0230536 (PMC7098575; doi:10.1371/journal.pone.0230536)
Supplement: Attachment — Submitted filename: lopez_garcia_et_al_IWANN_Response_Reviewers.pdf [file pone.0230536.s005.pdf]

PONE-D-19-33176
Transfer learning with convolutional neural networks for cancer survival prediction using gene-expression data
PLOS ONE

We would like to thank the editor and the reviewers for their fair comments and suggestions and for giving us the opportunity to improve and to re-submit our paper. In this revised version of our paper we have addressed all the comments pointed out by the reviewers and emphasised the novel contributions beyond our previous related work.

COMMENTS FOR REVIEWER #1:

We would like to thank this reviewer for her/his comments and suggestions.

The class imbalance problem should be handled in different ways for obtained unbiased results. Under sampling, over sampling and ensemble techniques do exist for performing this task.

As it has been fairly suggested by this reviewer, we conducted additional experiments in order to handle the severe class imbalance of the gene-expression datasets. Different techniques—random under-sampling, SMOTE and random over-sampling—were employed to re-sample the data before feeding it to the predictive models. We analyzed the performance of the models using each technique, and finally selected the one that maximized the mean CV AUC metric. In this way, random over-sampling procedure applied both during pre-training and fine-tuning phases of the TL approach gave the best results both using CNN and MLNN models. When applied in combination with the traditional ML approach, SMOTE gave the highest AUC values. The revised version of the paper has been updated with the new results obtained using random-oversampling and SMOTE in combination with DL-with-TL approach and the traditional ML approach, respectively. Figure 8 has also been updated.

The figures need to be updated in terms of resolution, they are not in the preferred format I think.

Following the submission guidelines, all figures were uploaded as separate files. Besides, we used PACE software, as suggested in Figures guidelines, to ensure all figures met PLOS requirements.

A comparison table with similar studies-methodologies should be presented in order to clearly highlight the impact of the current methodology and its usage in another studies.

To the best of our knowledge, we are the first authors to explore a TL approach using CNN models on gene-expression data to tackle a cancer-survival predictive task. For this reason, the results obtained in this paper are not directly comparable with the results achieved in previous works, as different methodologies are applied as well as completely distinct cancer

prediction tasks are tackled. However, we totally agree with this reviewer on the necessity of performing a thorough comparison between our proposed methodology and similar-studies methodologies to lucidly emphasize the impact of our contribution. In this way, the final paragraphs of the *Results and Discussion* section in the revised version of the manuscript addresses this concern.

COMMENTS FOR REVIEWER #2:

We would like to thank this reviewer for her/his comments and suggestions.

Authors can add information about the result of the selection of features using applied FS methods. How many original features were considered significant.

We considered the number of input features to be selected by the FS method as a hyper-parameter of the traditional ML approach. The range of possible values of this hyper-parameter, as well as the possible numbers of features to be extracted by the feature extraction methods, are described in detail in S3 Table.

Maybe also describing the technical aspects of the algorithm implementation might be of interest to readers. Methods must be described in sufficient detail for another researcher to reproduce the experiments described.

As stated in the revised version of the paper, for reproducing the results obtained in this work, all the data, code and technical descriptions of the algorithm implementation have been made publicly available at https://github.com/guilopgar/GeneExpImgTL
In addition, the Supporting Information file contains a detailed description of the hyper-parameter optimization procedure employed to perform automatic tuning of the hyper-parameters of all the models analyzed in this work.

Authors should provide adequate justification that the methods presented in the paper are significantly different from previously presented.

As it has been fairly suggested by this reviewer, the *Results and Discussion* section from the revised version of the manuscript contains a detailed description of the novelties and methodological contributions of the current study, as well as a thorough comparison between our proposed methodology and other similar-studies methodologies.
